# Plasma calprotectin was associated with platelet activation and no-reflow phenomenon in acute coronary syndrome

**DOI:** 10.1186/s12872-020-01717-5

**Published:** 2020-10-09

**Authors:** Nian-Peng Song, Xiao-Wen Zhen, Liu-dong Li, Lin Zhong, Hua Wang, Yi An

**Affiliations:** 1 Affiliated Hospital of Qingdao University, Qingdao University, Qingdao, China; 2 Department of Cardiology, Affiliated Yantai Yuhuangding Hospital of Qingdao University, Qingdao University, Yantai, China; 3 Department of Diagnostics, BinZhou Medical University, Yantai, China

**Keywords:** Calprotectin, Platelet activation, No-reflow, Acute coronary syndrome

## Abstract

**Background:**

No-reflow occurs in 3–4% of all percutaneous coronary interventions (PCIs) and has a strong negative impact on clinical outcomes of acute coronary syndrome (ACS). Therefore, the discovery of a biomarker that can early predict the occurrence of no-reflow has great clinical significance. Multiple factors including platelet activation are relevant to no-reflow. Calprotectin is found to be a biomarker of plaque instability and is identified to be a novel diagnostic and prognostic biomarker of cardiovascular diseases. The association of plasma calprotectin with platelet activation and no-reflow phenomenon in ACS is not clear.

**Methods:**

In this prospective study performed at Yantai Yuhuangding Hospital from 2017 to 2018, a total of 176 Chinese patients with ACS who had undergone PCIs were recruited consecutively, aged from 30 to 88 years. Angiographic no-reflow was defined as thrombolysis in myocardial infarction grade less than 3. Blood samples were collected immediately at admission for the detection of plasma calprotectin and platelet–monocyte aggregates formation. Statistical analysis was performed for the variable’s comparisons between groups and the prediction value of plasma calprotectin for no-reflow.

**Results:**

The mean age of the 176 included ACS patients were 64(±11) years and acute ST-segment elevation myocardial infarction (STEMI) was present in 41.5% of patients. Twenty-two patients had no-reflow during the PCI procedures and the prevalence was 12.5%. Patients with higher plasma calprotectin had a higher level of platelet–monocyte aggregates (PMA) and a higher prevalence of no-reflow (*p* < 0.001). The multivariate regression showed that plasma calprotectin and admission hs-cTnI were independently associated with PMA, while plasma calprotectin and serum LDL-c were independent predictors of no-reflow (*p* < 0.001 and *p* = 0.017). AUC of calprotectin for predicting no-reflow were 0.898. The cut-off value of plasma calprotectin for no-reflow was 4748.77 ng/mL with a sensitivity of 0.95 and a specificity of 0.77.

**Conclusion:**

Plasma calprotectin was associated with platelet activation and may act as an early predictive biomarker of no-reflow in patients with acute coronary syndrome.

## Background

No-reflow can occur in all percutaneous coronary interventions (PCIs), especially in emergency PCIs, and has a strong negative impact on clinical outcomes of acute coronary syndrome (ACS). Indeed, patients with no-reflow exhibit a higher prevalence of mortality, heart failure, and early postinfarction complications [1–3]. Consequently, early detection and appropriate prevention strategies of no-reflow have an important impact on the outcome of ACS.

Calprotectin, a heterotetramer of proteins S100A8 and S100A9, is identified to be a novel diagnostic and prognostic biomarker of cardiovascular diseases [4]. Calprotectin increases in the high-risk unstable or vulnerable atherosclerotic plaques in coronary arteries [5]. Increasing plasma calprotectin was associated with a higher risk of a recurrent cardiovascular event and significantly increased risk of cardiovascular death or myocardial infarction in ACS patients [6, 7]. Besides, circulating calprotectin is associated with thromboxane-dependent platelet activation in ACS [8]. Higher platelet reactivity and activation were found to be associated with an elevated prevalence of no-reflow after PCI in ACS patients [9].

Despite these studies, the association of plasma calprotectin with platelet activation and no-reflow phenomenon in ACS is not clear. The objective of this study was to investigate the relationship between calprotectin and platelet activation and evaluate the value of plasma calprotectin in predicting the development of a no-reflow phenomenon in ACS patients.

## Methods

### Study population

This prospective study was performed at Yantai Yuhuangding Hospital. A total of 176 Chinese patients with ACS who had undergone PCI were recruited consecutively from 2017 to 2018, aged from 30 to 88 years. ACS diagnosis criteria were defined according to published guidelines [10, 11]. Patients with a history of chronic kidney disease, inflammatory bowel disease, malignancy, severe infection, significant hepatic dysfunction, and auto-immune diseases were excluded. The study protocol was approved by the local institutional ethics committee. All patients provided written consent and received standard treatment according to the ACS management guidelines. Permission was granted to use data for analysis.

### Data collection and variable definitions

Demographic and clinical data were captured for all patients. The coronary angiography and PCI procedures were performed according to the current standard practice [12]. The data during the PCI procedures including number of diseased vessels, stent implantation rate, stent count per patient, stent length, stent diameter, thrombus aspiration, and intra-aortic balloon pump was collected. The Thrombolysis In Myocardial Infarction (TIMI) scoring systems were applied to evaluate the anterograde flow in the target culprit coronary artery and angiographic no-reflow was defined as a TIMI grade less than 3 [13, 14].

Other outcomes of interest included in-hospital major adverse cardiac events (MACE), including death, non-fatal myocardial infarctions, acute heart failure, chest pain, complete atrioventricular block, ventricular fibrillation, and ventricular tachycardia.

### Biomarker assays

Blood samples were collected immediately at admission before coronary angiography. Whole blood was carefully drawn via peripheral venipuncture into sterile acid-citrate-dextrose vacutainer tubes. The first 5mLs of blood were discarded. The remaining blood was immediately transported at room temperature to the laboratory for the detection of platelet–monocyte aggregates (PMA) formation with whole blood flow cytometry. The plasma was frozen at − 80 °C for further analysis. Plasma calprotectin was measured using an enzyme-linked immunosorbent assay kit (Biolegend, USA).

### Statistical analysis

The continuous variable data were tested for normality distribution with the Kolmogorov-Smirnov test and normal distribution parameters were presented as the mean ± standard deviation (SD). The independent-sample t-test and the Mann-Whitney U test were used for comparison of the study groups. Categorical variables were compared using Pearson’s chi-square test or Fisher’s test and presented as absolute counts and percentages. To determine the association among variable biomarkers and platelet activation, the Pearson correlation analyses, and multivariate linear regression analyses were performed. The predictive parameters for no-reflow were assessed using logistic regression analysis, and the variables with a *p*-value of < 0.1 were included in multivariate analysis for no-reflow by using a multiple logistic regression model. The discrimination power of calprotectin for no-reflow was assessed using the receiver operating characteristic curve (ROC). The area under the receiver operating characteristic curve (AUC) analysis calculated cut-off values, sensitivity, and specificity. A *p*-value of < 0.05 was considered statistically significant. Data analysis was performed using SPSS version 22 (SPSS Inc., Chicago, IL, USA).

## Results

### ACS patients with lower plasma calprotectin level versus ACS patients with higher plasma calprotectin level

A total of 176 ACS patients were included in this study and the patients were divided into 2 groups according to the median of calprotectin detected in our study (3681 ng/mL). There were 83 patients (mean age 63 ± 10 and 65.1% male) in the lower calprotectin group (group 1) and 93 patients (mean age 65 ± 12 and 76.1% male) in the higher calprotectin group (group 2). Baseline demographic, clinical, laboratory and procedure characteristics are shown in Table 1. The mean age, gender, history of smoking, hypertension, hypercholesterolemia and previous medication history did not differ. More absolute number of in-hospital mortality and in-hospital MACE was found in patients with higher calprotectin, but it did not differ significantly. Patients with higher calprotectin had a higher percentage of STEMI and higher GRACE scores (*p* < 0.001). Concerning coronary risk factors, there was a significantly higher presence of diabetes mellitus (*p* = 0.012) in patients with higher calprotectin.

**Table 1:** Comparison of variables between groups divided according to the median of calprotectin

variables	calprotectin <3681 ng/mL	calprotectin >3681 ng/mL	*P* value
	*N* = 83	*N* = 93	
Age (y)	63 ± 10	65 ± 12	0.228
Male, n (%)	54 (65.1)	70 (76.1)	0.109
Smoking, n (%)	18 (21.7)	23 (25.0)	0.605
Hypertension, n (%)	36 (43.4)	53 (57.6)	0.060
Diabetes mellitus, n (%)	12 (14.5)	28 (30.4)	0.012
Hypercholesterolemia, n (%)	24 (28.9)	35 (37.6)	0.221
STEMI, n (%)	15 (18.1)	58 (63.0)	<0.001
Body mass index (kg/m^2^)	26.4 ± 2.4	27.3 ± 2.6	0.862
TC (mmol/L)	4.6 ± 1.07	4.99 ± 1.3	0.037
LDL-c (mmol/L)	2.65 ± 0.88	3.04 ± 1.02	0.007
HDL-c (mmol/L)	1.12 ± 0.25	1.13 ± 0.24	0.911
Triglyceride (mmol/L)	1.37 ± 0.62	1.38 ± 0.83	0.962
Cystin-c (mg/L)	0.92 ± 0.17	1.04 ± 0.36	0.092
Glucose on admission (mmol/L)	8.60 ± 5.51	11.17 ± 5.78	0.009
Hs-cTnI on admission (pg/mL)	3020.70 ± 10,864.68	12,180.81 ± 18,134.70	<0.001
BNP on admission (pg/mL)	134.29 ± 207.14	419.02 ± 618.32	<0.001
Serum creatine (umol/L)	69.20 ± 15.02	86.40 ± 44.87	0.001
Blood urea nitrogen (mmol/L)	5.54 ± 1.65	6.67 ± 4.27	0.026
Fasting blood glucose (mmol/L)	6.60 ± 2.97	7.66 ± 2.85	0.018
CRP (mg/L)	8.00 ± 8.52	38.63 ± 55.03	0.005
WBC (*10^9^/L)	7.79 ± 3.82	9.57 ± 3.88	0.003
Neutrophil count (*10^9^/L)	5.23 ± 3.45	7.00 ± 3.65	0.001
N/L ratio	3.37 ± 2.84	5.41 ± 3.5	<0.001
Hemoglobin (g/L)	139.83 ± 15.94	141.4 ± 22.28	0.596
Platelet (*10^9^/L)	230.85 ± 87.43	231.87 ± 71.82	0.933
MPV (fl)	9.88 ± 1.81	9.51 ± 2.60	0.280
PDW (fl)	11.88 ± 3.51	11.57 ± 1.98	0.479
D-Dimer (mg/L)	0.64 ± 0.35	0.96 ± 0.89	0.002
LVEF (%)	60.53 ± 6.45	57.37 ± 7.8	0.004
LVDd (mm)	44.9 ± 6.06	47.97 ± 5.53	0.005
GRACE score	102.23 ± 36.27	139.03 ± 36.73	<0.001
PMA (%)	32.43 ± 11.05	43.2 ± 12.53	<0.001
Number of diseased vessels	1.46 ± 1.03	1.68 ± 0.96	0.062
Stent implantation, n (%)	75 (90.4)	86 (92.5)	0.616
Total stent count per patient, n	1.72 ± 1.20	1.83 ± 1.36	0.245
Total stent length (mm)	23.42 ± 9.56	24.78 ± 10.83	0.183
Stent diameter (mm)	3.13 ± 0.30	3.24 ± 0.32	0.876
Thrombus aspiration, n (%)	5 (6.0)	15 (16.1)	0.029
Intra-aortic balloon pump, n (%)	3 (3.6)	10 (10.8)	0.062
Plasma calprotectin (ng/mL)	2489.72 ± 747.58	5233.32 ± 1125.62	<0.001
Discharge BNP (pg/mL)	123.44 ± 185.55	485.55 ± 601.63	<0.001
Albumin (g/L)	39.11 ± 3.59	37.60 ± 3.85	0.008
Previous CAD, n (%)	29 (34.9)	32 (34.4)	0.941
Medications previous, n (%)			
Aspirin	28 (33.7)	30 (32.3)	0.835
Glycoprotein IIb/IIIa antagonist	10 (12.0)	12 (12.9)	0.864
ACEI	21 (25.3)	24 (25.8)	0.939
B-blocker	23 (27.7)	18 (19.4)	0.190
Statin	31 (37.3)	36 (38.7)	0.853
Nitrate	20 (24.1)	27 (29.0)	0.460
No-reflow, n (%)	1 (1.2)	21 (22.8)	<0.001
In-hospital MACE, n (%)	3 (3.6)	8 (8.6)	0.172
In-hospital mortality, n (%)	1 (1.2)	4 (4.3)	0.217

Values are expressed as mean ± SD (standard deviation) or number (%)

*STEMI* Acute ST-segment elevation myocardial infarction; *TC* Total cholesterol; *LDL-c* Low-density lipoprotein cholesterol; *HDL-c* High-density lipoprotein cholesterol; *BNP* B-type natriuretic peptide; *hs-cTnI* High-sensitive cardiac troponin I; *CRP* C-reactive protein; *WBC* White blood cell count; *N/L ratio* Neutrophil-lymphocyte ratio; *MPV* Mean platelet volume; *PDW* Platelet distribution width; *LVEF* Left ventricle ejection fraction; *LVDd* Left ventricular diastolic diameter; *PMA* Platelet–monocyte aggregates; *CAD* Coronary artery disease; *ACEI* Angiotensin-converting-enzyme inhibitor; *MACE* Major adverse cardiac events

For the baseline laboratory variables, patients with higher calprotectin were shown to have a higher level of the total cholesterol (TC), low-density lipoprotein cholesterol (LDL-c), admission serum glucose, and fasting blood glucose (all *p* < 0.05). Compared with patients with lower calprotectin, patients with higher calprotectin had a significantly higher level of admission high-sensitive cardiac troponin I (hs-cTnI) and B-type natriuretic peptide (BNP) (all *p* < 0.001). In addition, patients with higher calprotectin seemed to have worse renal function and higher inflammation level (all *p* < 0.05). As for echocardiography variables, significantly higher left ventricular diastolic diameter (LVDd) and lower left ventricular ejection fraction (LVEF) were found in patients with higher calprotectin (*p* = 0.005 and *p* = 0.004) (Table 1).

Concerning the procedural characteristics, patients with higher plasma calprotectin were more likely to have a higher number of diseased vessels, be treated with longer and greater diameter stents, and have higher usage of intra-aortic balloon pump, but the difference was not significant (all *p* > 0.05). A higher incidence of thrombus aspiration was present in patients with higher plasma calprotectin (*p* = 0.029). Besides, platelet activation biomarker PMA was found to be significantly higher in patients with higher plasma calprotectin (*p* < 0.001) (Table 1).

### ACS patients with no-reflow versus ACS patients without no-reflow

**Fig. 1:**
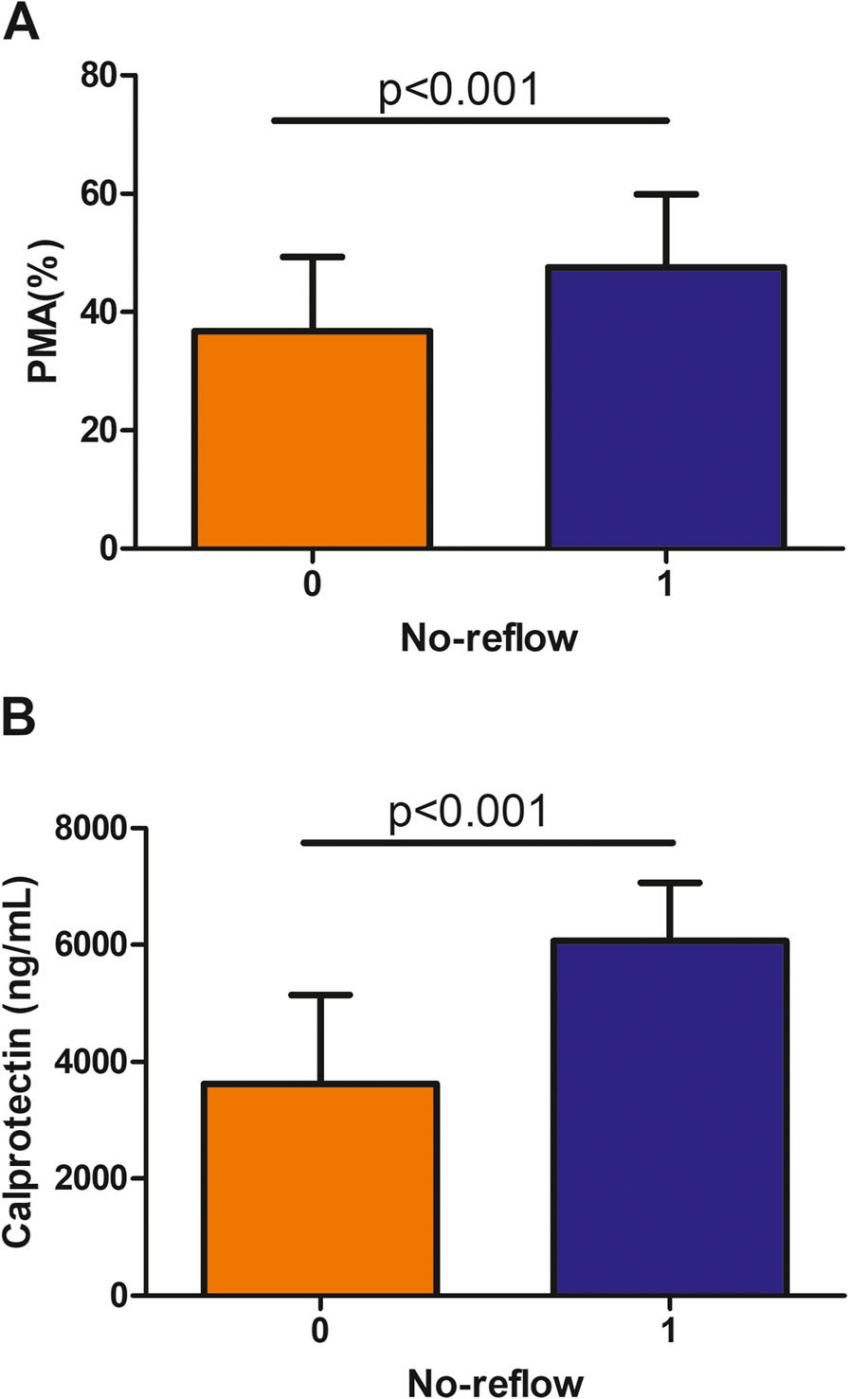
Comparison of calprotectin and PMA between ACS patients with and without no-reflow. **a** ACS patients with no-reflow had higher PMA (47.58 ± 12.30% vs 36.73 ± 12.55%, ***p < 0.001) and (**b**) higher plasma calprotectin (6062.9 ± 999.8 vs 3625.7 ± 1526.8 ng/mL, ***p < 0.001) compared with those without no-reflow. Data are means ± SD

ACS patients with no-reflow had higher plasma calprotectin and PMA compared with those without no-reflow (6062.9 ± 999.8 vs 3625.7 ± 1526.8 ng/mL, *p* < 0.001; 47.58 ± 12.30% vs 36.73 ± 12.55%, *p* < 0.001, respectively) as shown in Fig. 1 (a and b).

### Determinants of plasma calprotectin and PMA in ACS patients

**Fig. 2:**
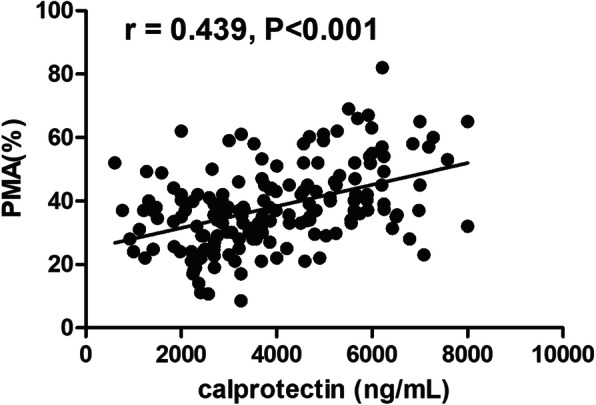
Correlation between plasma calprotectin and PMA in ACS patients. Plasma calprotectin was positively correlated with PMA (*r* = 0.439, *p* < 0.001)

The analyses of correlation demonstrated that either calprotectin or PMA were positively associated with hs-cTnI on admission, BNP on admission, glucose on admission, GRACE score, LDL-c, TC, CRP, WBC, and neutrophil-lymphocyte ratio (N/L). Calprotectin and PMA were negatively associated with LVEF (Table 2). Figure 2 showed that calprotectin was positively correlated with PMA (*r* = 0.439, *p* < 0.001). Only calprotectin and hs-cTnI on admission were found to be independently associated with PMA as shown in Table 3.

**Table 2:** Correlations of baseline laboratory factors with calprotectin and PMA

variables	calprotectin		PMA	
	r	*P*	r	*p*
hs-cTnI on admission	0.335	<0.001	0.367	<0.001
BNP on admission	0.297	<0.001	0.236	0.002
GRACE score	0.445	<0.001	0.385	<0.001
LVEF	−0.240	0.001	−0.205	0.006
LDL-c	0.264	<0.001	0.252	0.001
TC	0.240	0.002	0.192	0.012
CRP	0.472	<0.001	0.248	0.030
WBC	0.358	<0.001	0.377	<0.001
N/L ratio	0.322	<0.001	0.295	<0.001
Glucose on admission	0.256	0.002	0.191	0.024

**Table 3:** Multivariate linear regression analysis of various laboratory factors and PMA

model		Unstandardized Coefficients		Standardized Coefficients	T	*P* value
		B	Standard error	β		
1	calprotectin	0.0019	0.0007	0.25	2.78	0.006
	hs-cTnI on admission	0.00018	0.00007	0.24	2.70	0.008
	BNP on admission	0.003	0.0020	0.12	1.48	0.143
	LDL-c	1.36	1.01	0.10	1.35	0.180
	WBC	0.26	0.28	0.08	0.92	0.359
	Glucose on admission	0.23	0.18	0.10	1.29	0.199

### Determinants of no-reflow in ACS patients

**Fig. 3:**
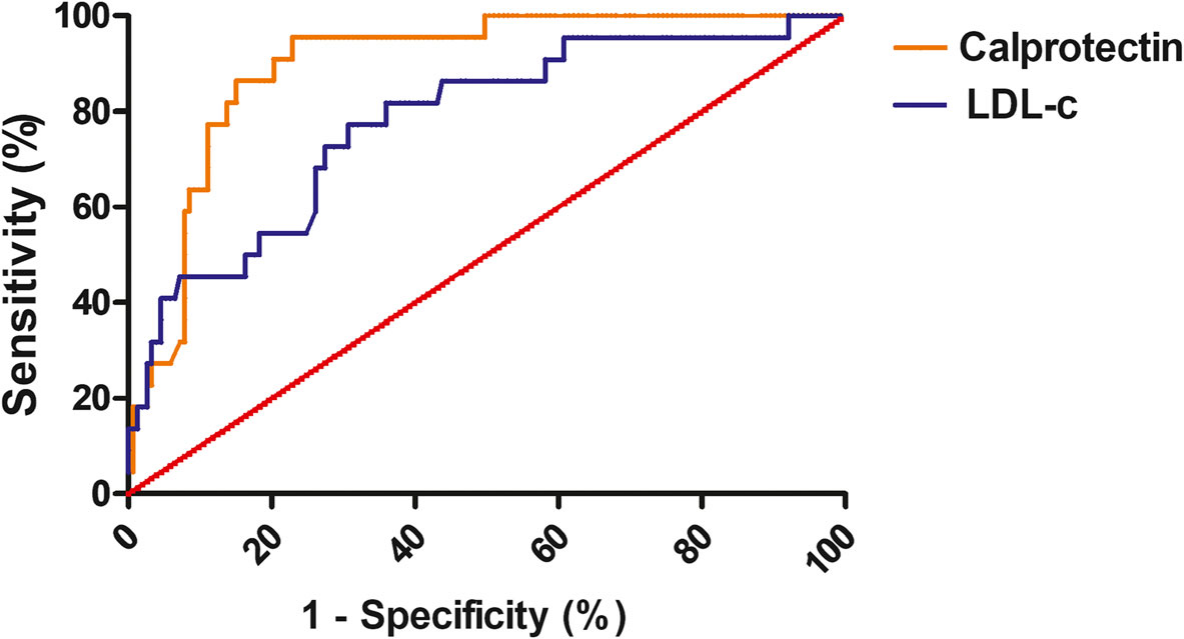
The ROC curves of calprotectin and LDL-c for predicting no-reflow in ACS patients. AUC of calprotectin and LDL-c for predicting no-reflow were 0.898 and 0.779, respectively

The analysis of univariate logistic regression revealed that no-reflow was associated with calprotectin, PMA, diabetes mellitus history, LDL-c, N/L ratio, glucose on admission, and BNP on admission (all *p* < 0.05). The multivariate logistic regression of the variables with an unadjusted *p* < 0.10 showed that only calprotectin and LDL-c were independent predictors of no-reflow (*p* < 0.001 and *p* = 0.006 respectively) (Table 4). The ROC curves of calprotectin and LDL-c for predicting no-reflow were shown in Fig. 3. AUC of calprotectin and LDL-c for predicting no-reflow were 0.898 and 0.779, respectively. The cut-off value of plasma calprotectin for no-reflow was 4748.77 ng/mL with a sensitivity of 0.95 and a specificity of 0.77. The cut-off value of LDL-c for no-reflow was 3.06 mmol/L with a sensitivity of 0.77 and a specificity of 0.69.

**Table 4:** Univariate and multivariate logistic regression analyses of multiple variables and the no-reflow

variables	Unadjusted OR	*p* value	Adjusted OR	95% CI	*p* value
calprotectin	1.001	<0.001	1.001	1.001–1.002	<0.001
PMA	1.064	<0.001	0.996	0.942–1.053	0.887
Diabetes millitus	5.357	<0.001	2.222	0.450–10.979	0.327
LDL-c	3.042	<0.001	3.250	1.401–7.539	0.006
WBC	1.142	0.007	0.877	0.677–1.136	0.319
N/L ratio	1.169	0.008	1.143	0.892–1.465	0.290
Glucose on admission	1.083	0.025	1.045	0.838–1.303	0.694
BNP on admission	1.001	0.024	1.000	0.999–1.001	0.590
Age	0.990	0.621			
Male	0.865	0.768			
aspirine	0.663	0.369			
smoking	0.956	0.934			
hs-cTnI on admission	1.000	0.076	1.000	1.000–1.000	0.559
CRP	1.010	0.057	0.990	0.972–1.008	0.258
D-Dimer	1.101	0.749			
MPV	0.952	0.577

## Discussion

In this study, we have demonstrated that ACS patients with higher plasma calprotectin had an elevated level of platelet activation and a higher incidence of no-reflow. The plasma calprotectin level was independently associated with platelet activation and no-reflow in patients with ACS. Despite that platelet activation biomarker PMA was associated with no-flow, only plasma calprotectin and serum LDL-c acted as independent predictors of no-reflow in patients with ACS as shown in the present study.

In humans, no-reflow may occur in emergency PCI or elective PCI for ACS. The occurrence of no-reflow after PCI decreased the efficacy of reperfusion therapy and is associated with worse clinical outcomes [15]. Following primary PCI for acute myocardial infarction (AMI), no-reflow measured by angiography remarkably increases the short-term mortality risk at 30 days [2] and long-term mortality risk at 1 year [16, 17] and 5 years respectively [18]. Therefore, the discovery of a biomarker that can early predict the occurrence of no-reflow has great clinical significance.

Calprotectin is an inflammation-associated peptide with proinflammatory properties, mainly secreted from activated neutrophils and monocytes under various conditions [4]. Calprotectin is traditionally thought to be involved in the pathophysiology of various inflammatory conditions such as rheumatoid arthritis [19]. However, some recent studies implied that calprotectin may be implicated in the pathogenesis of cardiovascular and cardiometabolic diseases based on low-grade chronic inflammation [6, 20].

High levels of calprotectin were found in human atherosclerotic plaques and it is correlated with the characteristics of rupture-prone lesions [5]. As a result, calprotectin is supposed to be a biomarker of plaque instability [21]. Calprotectin is also found to be specifically expressed in neutrophils and macrophages in infarcted myocardium [22]. Blood calprotectin levels are markedly higher in ACS patients than in stable CAD or healthy subjects [20, 23] and plasma levels of calprotectin were significantly elevated in patients with AMI than in patients with unstable angina pectoris [22]. Moreover, levels of calprotectin were also found to be higher in STEMI patients who died after a median 12 months follow-up compared to the STEMI patients who survived [7]. Calprotectin has been associated with an increased risk of cardiovascular death or myocardial infarction after ACS [6]. What’s more, calprotectin is found to be correlated with first and recurrent cardiovascular events in middle-aged healthy individuals [24]. Similarly, our study revealed that calprotectin was positively correlated with admission cTnI, BNP, and GRACE score. A negative association between calprotectin and LVEF was also present in our study.

Despite the important roles of calprotectin in ACS, the role of calprotectin in the no-reflow phenomenon of ACS patients has not been clarified. The pathophysiology of post-PCI no-reflow is complex and it involves inflammation, vasoconstriction, higher platelet reactivity, and microcirculation embolization [25]. Micro-embolization in distal coronary circulation may occur after plaque rupture or erosion and subsequent thrombosis. Thrombotic material from the originally occurred thrombus may move distally and embolize smaller arteries, thus causing no-reflow. Increased calprotectin concentration was found in aspirated coronary artery blood distal to the culprit ACS lesion associated with thrombosis and is related to local leukocyte activation. Thus, calprotectin is supposed to be a biomarker of inflammation and thrombosis in ACS [26]. In the current study, we firstly demonstrated that calprotectin was an independent predictor of no-reflow in ACS patients.

Cardiovascular risk factors, such as smoking, hypercholesterolemia, diabetes mellitus, and other inflammatory biomarkers are thought to be conventional risk predictors for no-reflow [27, 28]. Diabetes mellitus and hypercholesterolemia are the main risk factor of ACS and is associated with coronary thrombosis, microvascular dysfunction and inflammation processes [29]. In the present study, we demonstrated that plasma calprotectin was related to these risk factors such as admission glucose, LDL-c, WBC, and N/L ratio. Besides, diabetes mellitus, admission glucose, LDL-c, WBC, and N/L ratio were predictors of no-reflow consistent with previous studies [25, 27, 28, 30]. These findings explain to some extent why plasma calprotectin can act as a predictor of no-reflow.

LDL-c was an independent predictor of no-reflow with lower sensitivity and specificity compared with calprotectin in ACS patients as shown in the current study. LDL-c plays a fundamental role in the pathophysiology of CAD. By now, it is well known that the property of atherosclerotic plaques may determine their thrombogenicity [31]. Vulnerable plaques like lipid-rich plaques with thin caps are more likely to form thrombus than stable plaques with thick caps and poor lipid cores [32]. Erosion or rupture of a vulnerable plaque directly activates platelets and causing thrombus formation by the exposure of thrombogenic materials including collagens and a lipid-rich atheromatous core comprising of oxidized LDL particles and cholesterol sulfate. It has been confirmed by intra-coronary imaging that the lipid-rich and necrotic core rich culprit plaques may act as an important predictor of distal embolization and no-reflow in ACS patients [33]. Compared with normocholesterolemic rabbits, hypercholesterolemic rabbits demonstrated markedly increased no-reflow [34]. Patients undergoing elective PCI with preprocedural statin therapy have a decreased incidence of periprocedural myocardial infarction compared with that in patients with no statin therapy [35]. In patients with AMI, long-term use of statins improved coronary flow and reduced the incidence of no-reflow [36]. White blood cell subtypes play crucial roles in modulating the inflammation in the atherosclerotic process and N/L ratio is thought to be an independent predictor of no-reflow after primary PCI [30]. In the present study, we also found that WBC and N/L ratios were associated with no-reflow. Moreover, WBC and N/L ratios were positively correlated with calprotectin and PMA. Some studies have confirmed neutrophil activation and accumulation in the myocardial area affected by acute coronary occlusion [37]. This accumulation is further increased after reperfusion and is another potential source of free radicals [37]. Interaction between activated neutrophils and damaged endothelium may induce endothelial dysfunction and vasoconstriction [38]. Inhibition of selectin adhesion molecules influencing the interaction between activated neutrophils and damaged endothelium has been shown to limit infarct size in animal models [39].

The essential role of platelets for the pathogenic thrombosis development in ACS is highlighted by a large body of evidence. There are increased plasma concentrations of indicators of platelet activation in patients with ACS compared to those with stable CAD or normal populations [40, 41]. Platelet magnifies chronic inflammation and interaction of platelet with leukocytes, endothelial cells and macrophages promote a proinflammatory and prothrombotic setting leading to plaque instability and subsequent intracoronary thrombosis [42]. Platelets may induce inflammatory reactions directly and indirectly by promoting inflammation and recruitment of inflammatory cells. Platelets adhere to the endothelium of small coronary arteries get activated and release several leukocyte recruitment molecules and vasoactive molecules [43]. For these reasons, platelets contribute to ACS not only by inducing the intraluminal thrombosis but also by micro-embolization in the distal coronary circulation, by local thrombosis and vasoconstriction in the microcirculation, and by platelet-mediated inflammatory reactions [44]. Accordingly, higher platelet reactivity and activation were found to be associated with an elevated prevalence of no-reflow after PCI in ACS patients [9]. Correlation between plasma calprotectin and thromboxane-dependent platelet activation has been demonstrated in ACS patients [8]. In the current study, we also found that plasma calprotectin was positively correlated with platelet activation biomarker PMA in ACS patients and PMA was positively correlated with no-reflow in ACS patients.

In this study, we demonstrated that ACS patients with higher plasma calprotectin had a higher incidence of no-reflow and plasma calprotectin might act as an independent predictor of no-reflow in patients with ACS. The mechanism of no-reflow seems to imply many pathways and probably only a part has been clarified. Further basic researches are needed to better understand the specific mechanism of calprotectin in the development of no-reflow.

Our study has some limitations. It was a single-center, prospectively designed study with relatively small sample size. Bias may exist and the findings should be interpreted cautiously. We detected only an admission blood sample of plasma calprotectin with no information about the temporal trend of changes. Optimal predictive cut-off levels and the predictive performance of plasma calprotectin for no-reflow in ACS patients will require validation in a larger scale, prospective, multi-center studies.

## Conclusion

Plasma calprotectin was associated with platelet activation and may act as an early predictive biomarker of no-reflow in patients with acute coronary syndrome. Our findings should be further confirmed in future multicenter, prospectively designed studies.

## Data Availability

The datasets used and/or analyzed during the current study available from the corresponding author on reasonable request.

## Abbreviations

PCI: Percutaneous coronary intervention
ACS: Acute coronary syndrome
TIMI: Thrombolysis in myocardial infarction
MACE: Major adverse cardiac events
PMA: Platelet–monocyte aggregates
SD: Standard deviation
ROC: Receiver operating characteristic curve
AUC: Area under the receiver operating characteristic curve
STEMI: ST-segment elevation myocardial infarction
CAD: Coronary artery disease
TC: Total cholesterol
LDL-c: Low-density lipoprotein cholesterol
HDL-c: High-density lipoprotein cholesterol
MPV: Mean platelet volume
PDW: Platelet distribution width
Hs-cTnI: High-sensitive cardiac troponin I
BNP: B-type natriuretic peptide
CRP: C-reactive protein
WBC: White blood cell count
LVDd: Left ventricular diastolic diameter
LVEF: Left ventricle ejection fraction
N/L: Neutrophil lymphocyte ratio
AMI: Acute myocardial infarction

## References

[1] Gibson CM, Cannon CP, Murphy SA, Marble SJ, Barron HV, Braunwald E (2002). Relationship of the TIMI myocardial perfusion grades, flow grades, frame count, and percutaneous coronary intervention to long-term outcomes after thrombolytic administration in acute myocardial infarction. Circulation.

[2] Bolognese L, Carrabba N, Parodi G, Santoro GM, Buonamici P, Cerisano G (2004). Impact of microvascular dysfunction on left ventricular remodeling and long-term clinical outcome after primary coronary angioplasty for acute myocardial infarction. Circulation..

[3] Brosh D, Assali AR, Mager A, Porter A, Hasdai D, Teplitsky I (2007). Effect of no-reflow during primary percutaneous coronary intervention for acute myocardial infarction on six-month mortality. Am J Cardiol.

[4] Montagnana M, Danese E, Lippi G (2014). Calprotectin and cardiovascular events. A narrative review. Clin Biochem.

[5] Ionita MG, Vink A, Dijke IE, Laman JD, Peeters W, van der Kraak PH (2009). High levels of myeloid-related protein 14 in human atherosclerotic plaques correlate with the characteristics of rupture-prone lesions. Arterioscler Thromb Vasc Biol.

[6] Morrow DA, Wang Y, Croce K, Sakuma M, Sabatine MS, Gao H (2008). Myeloid-related protein 8/14 and the risk of cardiovascular death or myocardial infarction after an acute coronary syndrome in the pravastatin or atorvastatin evaluation and infection therapy: thrombolysis in myocardial infarction (PROVE IT-TIMI 22) trial. Am Heart J.

[7] Jensen LJ, Pedersen S, Bjerre M, Mogelvang R, Jensen JS, Flyvbjerg A (2010). Plasma calprotectin predicts mortality in patients with ST segment elevation myocardial infarction treated with primary percutaneous coronary intervention. J Interv Cardiol.

[8] Santilli F, Paloscia L, Liani R, Di Nicola M, Di Marco M, Lattanzio S, et al. Circulating myeloid-related protein-8/14 is related to thromboxane-dependent platelet activation in patients with acute coronary syndrome, with and without ongoing low-dose aspirin treatment. J Am Heart Assoc. 2014;3(4). 10.1161/JAHA.114.000903 PMC4310373 25037196

[9] Campo G, Valgimigli M, Gemmati D, Percoco G, Tognazzo S, Cicchitelli G (2006). Value of platelet reactivity in predicting response to treatment and clinical outcome in patients undergoing primary coronary intervention: insights into the STRATEGY study. J Am Coll Cardiol.

[10] Rodriguez F, Mahaffey KW (2016). Management of Patients with NSTE-ACS: a comparison of the recent AHA/ACC and ESC guidelines. J Am Coll Cardiol.

[11] Ibanez B, James S, Agewall S, Antunes MJ, Bucciarelli-Ducci C, Bueno H (2018). 2017 ESC guidelines for the management of acute myocardial infarction in patients presenting with ST-segment elevation: the task force for the management of acute myocardial infarction in patients presenting with ST-segment elevation of the European Society of Cardiology (ESC). Eur Heart J.

[12] Levine GN, Bates ER, Blankenship JC, Bailey SR, Bittl JA, Cercek B (2011). 2011 ACCF/AHA/SCAI guideline for percutaneous coronary intervention: executive summary: a report of the American College of Cardiology Foundation/American Heart Association task force on practice guidelines and the Society for Cardiovascular Angiography and Interventions. Circulation.

[13] Ganz W (1985). The thrombolysis in myocardial infarction (TIMI) trial. N Engl J Med.

[14] van ‘t Hof AW, Liem A, Suryapranata H, Hoorntje JC, de Boer MJ, Zijlstra F. Angiographic assessment of myocardial reperfusion in patients treated with primary angioplasty for acute myocardial infarction: myocardial blush grade. Zwolle Myocardial Infarction Study Group. Circulation. 1998;97(23):2302–2306. 10.1161/01.cir.97.23.2302 9639373

[15] Resnic FS, Wainstein M, Lee MK, Behrendt D, Wainstein RV, Ohno-Machado L (2003). No-reflow is an independent predictor of death and myocardial infarction after percutaneous coronary intervention. Am Heart J.

[16] Sorajja P, Gersh BJ, Costantini C, McLaughlin MG, Zimetbaum P, Cox DA (2005). Combined prognostic utility of ST-segment recovery and myocardial blush after primary percutaneous coronary intervention in acute myocardial infarction. Eur Heart J.

[17] Henriques JP, Zijlstra F, van ‘t Hof AW, de Boer MJ, Dambrink JH, Gosselink M (2003). Angiographic assessment of reperfusion in acute myocardial infarction by myocardial blush grade. Circulation.

[18] Ndrepepa G, Tiroch K, Fusaro M, Keta D, Seyfarth M, Byrne RA (2010). 5-year prognostic value of no-reflow phenomenon after percutaneous coronary intervention in patients with acute myocardial infarction. J Am Coll Cardiol.

[19] de Seny D, Fillet M, Ribbens C, Marée R, Meuwis MA, Lutteri L (2008). Monomeric calgranulins measured by SELDI-TOF mass spectrometry and calprotectin measured by ELISA as biomarkers in arthritis. Clin Chem.

[20] Altwegg LA, Neidhart M, Hersberger M, Müller S, Eberli FR, Corti R (2007). Myeloid-related protein 8/14 complex is released by monocytes and granulocytes at the site of coronary occlusion: a novel, early, and sensitive marker of acute coronary syndromes. Eur Heart J.

[21] Schaub N, Reichlin T, Meune C, Twerenbold R, Haaf P, Hochholzer W (2012). Markers of plaque instability in the early diagnosis and risk stratification of acute myocardial infarction. Clin Chem.

[22] Katashima T, Naruko T, Terasaki F, Fujita M, Otsuka K, Murakami S (2010). Enhanced expression of the S100A8/A9 complex in acute myocardial infarction patients. Circ J.

[23] Vora AN, Bonaca MP, Ruff CT, Jarolim P, Murphy S, Croce K (2012). Diagnostic evaluation of the MRP-8/14 for the emergency assessment of chest pain. J Thromb Thrombolysis.

[24] Cotoi OS, Dunér P, Ko N, Hedblad B, Nilsson J, Björkbacka H (2014). Plasma S100A8/A9 correlates with blood neutrophil counts, traditional risk factors, and cardiovascular disease in middle-aged healthy individuals. Arterioscler Thromb Vasc Biol.

[25] Schwartz BG, Kloner RA (2012). Coronary no reflow. J Mol Cell Cardiol.

[26] Sakuma M, Tanaka A, Kotooka N, Hikichi Y, Toyoda S, Abe S (2017). Myeloid-related protein-8/14 in acute coronary syndrome. Int J Cardiol.

[27] Rezkalla SH, Stankowski RV, Hanna J, Kloner RA (2017). Management of no-Reflow Phenomenon in the catheterization laboratory. JACC Cardiovasc Interv.

[28] Zhang QY, Ma SM, Sun JY (2020). New CHA (2) DS (2)-VASc-HSF score predicts the no-reflow phenomenon after primary percutaneous coronary intervention in patients with ST-segment elevation myocardial infarction. BMC Cardiovasc Disord.

[29] Celik T, Kaya MG, Akpek M, Gunebakmaz O, Balta S, Sarli B (2015). Predictive value of admission platelet volume indices for in-hospital major adverse cardiovascular events in acute ST-segment elevation myocardial infarction. Angiology.

[30] Akpek M, Kaya MG, Lam YY, Sahin O, Elcik D, Celik T (2012). Relation of neutrophil/lymphocyte ratio to coronary flow to in-hospital major adverse cardiac events in patients with ST-elevated myocardial infarction undergoing primary coronary intervention. Am J Cardiol.

[31] Fuster V, Moreno PR, Fayad ZA, Corti R, Badimon JJ (2005). Atherothrombosis and high-risk plaque: part I: evolving concepts. J Am Coll Cardiol.

[32] Corti R, Farkouh ME, Badimon JJ (2002). The vulnerable plaque and acute coronary syndromes. Am J Med.

[33] Hong YJ, Jeong MH, Choi YH, Ko JS, Lee MG, Kang WY (2011). Impact of plaque components on no-reflow phenomenon after stent deployment in patients with acute coronary syndrome: a virtual histology-intravascular ultrasound analysis. Eur Heart J.

[34] Golino P, Maroko PR, Carew TE (1987). Efficacy of platelet depletion in counteracting the detrimental effect of acute hypercholesterolemia on infarct size and the no-reflow phenomenon in rabbits undergoing coronary artery occlusion-reperfusion. Circulation.

[35] Herrmann J, Lerman A, Baumgart D, Volbracht L, Schulz R, von Birgelen C (2002). Preprocedural statin medication reduces the extent of periprocedural non-Q-wave myocardial infarction. Circulation.

[36] Iwakura K, Ito H, Kawano S, Okamura A, Kurotobi T, Date M (2006). Chronic pre-treatment of statins is associated with the reduction of the no-reflow phenomenon in the patients with reperfused acute myocardial infarction. Eur Heart J.

[37] Seydoux C, Goy JJ, Davies G (1993). Platelet and neutrophil imaging techniques in the investigation of the response to thrombolytic therapy and the no-reflow phenomenon. Am Heart J.

[38] Murohara T, Buerke M, Lefer AM (1994). Polymorphonuclear leukocyte-induced vasocontraction and endothelial dysfunction. Role of selectins. Arterioscler Thromb.

[39] Birnbaum Y, Patterson M, Kloner RA (1997). The effect of CY1503, a sialyl Lewisx analog blocker of the selectin adhesion molecules, on infarct size and "no-reflow" in the rabbit model of acute myocardial infarction/reperfusion. J Mol Cell Cardiol.

[40] Heeschen C, Dimmeler S, Hamm CW, van den Brand MJ, Boersma E, Zeiher AM (2003). Soluble CD40 ligand in acute coronary syndromes. N Engl J Med.

[41] Bigalke B, Haap M, Stellos K, Geisler T, Seizer P, Kremmer E (2010). Platelet glycoprotein VI (GPVI) for early identification of acute coronary syndrome in patients with chest pain. Thromb Res.

[42] Gawaz M, Langer H, May AE (2005). Platelets in inflammation and atherogenesis. J Clin Invest.

[43] Gawaz M, Neumann FJ, Dickfeld T, Koch W, Laugwitz KL, Adelsberger H (1998). Activated platelets induce monocyte chemotactic protein-1 secretion and surface expression of intercellular adhesion molecule-1 on endothelial cells. Circulation..

[44] Stakos DA, Tziakas DN, Stellos K (2012). Mechanisms of platelet activation in acute coronary syndromes. Curr Vasc Pharmacol.

